# Inhalation of Chloroform for the Relief of Phymosis

**Published:** 1852-03

**Authors:** 


*Inhalation of Chloroform for the Relief of Phymosis.*—M. Guisard states *(L'Union Médicale,)* that his son, about three years of age, affected with phymosis, had been relieved from very distressing symptoms, by inhalation of chloroform. The little patient experienced great agony in passing urine, and the distress was so great that he overcame the necessity of performing this function, and obstinately refused to relieve his bladder.

He was placed under the influence of chloroform, so that the phymosed prepuce could be incised, and hardly had its effects taken place than the urine began to flow, and the rectum was emptied. The next day, the child was equally obstreperous, and the same means brought about the same results. M. Guisard thinks that these facts may open a new path for the treatment of retention of urine or of fecal matter.—*Ibid.*

